# Supplementary material for: Cumulative Weighing of Time in Intertemporal Tradeoffs
Source: J Exp Psychol Gen. 2016 Sep;145(9):1177–205. doi: 10.1037/xge0000198 (PMC4998108; doi:10.1037/xge0000198)
Supplement: Supplementary file 1 [file Scholten_Individuals.zip › plots/e31_p53_eg2_priors_and_posteriors.pdf]

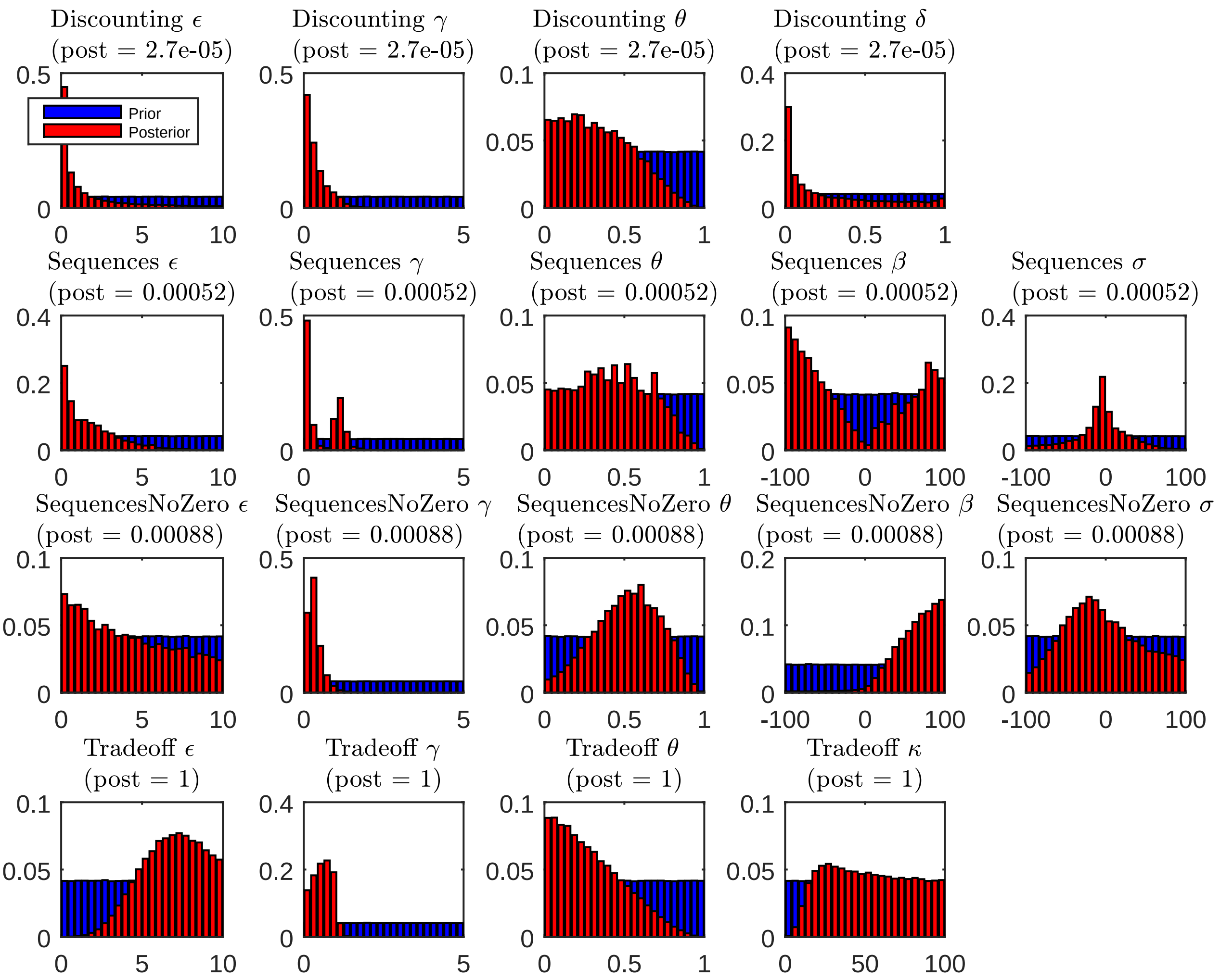
Discounting $\epsilon$ (post = 2.7e-05)
Discounting $\gamma$ (post = 2.7e-05)
Discounting $\theta$ (post = 2.7e-05)
Discounting $\delta$ (post = 2.7e-05)
Prior
Posterior
Sequences $\epsilon$ (post = 0.00052)
Sequences $\gamma$ (post = 0.00052)
Sequences $\theta$ (post = 0.00052)
Sequences $\beta$ (post = 0.00052)
Sequences $\sigma$ (post = 0.00052)
SequencesNoZero $\epsilon$ (post = 0.00088)
SequencesNoZero $\gamma$ (post = 0.00088)
SequencesNoZero $\theta$ (post = 0.00088)
SequencesNoZero $\beta$ (post = 0.00088)
SequencesNoZero $\sigma$ (post = 0.00088)
Tradeoff $\epsilon$ (post = 1)
Tradeoff $\gamma$ (post = 1)
Tradeoff $\theta$ (post = 1)
Tradeoff $\kappa$ (post = 1)
